# Poor Representation of Rural Counties of the United States in Some Measures of Consumer Broadband

**DOI:** 10.1089/tmr.2024.0048

**Published:** 2024-10-08

**Authors:** Cari A. Bogulski, Maysam Rabbani, Corey J. Hayes, Aysenur Betul Cengil, Catherine C. Shoults, Hari Eswaran

**Affiliations:** ^1^ Department of Biomedical Informatics, College of Medicine, University of Arkansas for Medical Sciences, Little Rock, Arkansas, USA.; ^2^ Institute for Digital Health and Innovation, University of Arkansas for Medical Sciences, Little Rock, Arkansas, USA.; ^3^ Feliciano School of Business, Montclair State University, Montclair, New Jersey, USA.; ^4^ Center for Mental Healthcare and Outcomes Research, Central Arkansas Veterans Healthcare System, North Little Rock, Arkansas, USA; ^5^ Department of Industrial Engineering, College of Engineering, University of Arkansas, Fayetteville, Arkansas, USA.; ^6^ Department of Obstetrics and Gynecology, College of Medicine, University of Arkansas for Medical Sciences, Little Rock, Arkansas, USA.

**Keywords:** broadband, internet, speed tests, digital divide, equity, rural

## Abstract

**Introduction:**

Telehealth has the potential to mitigate the lack of health care access in rural and underserved communities; however, telehealth is only viable where sufficiently high-speed internet broadband is available to patients. Existing broadband data sets may not accurately reflect the state of broadband, particularly in rural communities. We examined consumer internet speed test data from two organizations to see if the number of tests per 1,000 residents varied across county-level rurality.

**Methods:**

We analyzed county-level data from Measurement Labs (M-Lab) and Ookla for Good (Ookla fixed and mobile) across the calendar years 2020 and 2021. We used the number of tests conducted per 1,000 residents within United States counties as the outcome variable, and Rural-Urban Continuum Codes (RUCC) as the main independent variable of interest.

**Results:**

Using negative binomial models with robust standard errors, we found that the number of fixed speed tests conducted per 1,000 residents was generally lower in rural counties relative to counties with over one million residents. However, we found no associations between any categories of county-level rurality for the number of mobile tests conducted per 1,000 residents. Patterns of association with other covariates emerged as significant in some models and not in others, suggesting key differences among users generating speed tests among these data sources.

**Conclusions:**

Our findings demonstrate the poor representation of residents from very rural counties in M-Lab and Ookla fixed data sets of user-generated internet speed tests. Additional data are needed to inform broadband infrastructure investment to identify those communities most left behind by broadband expansion efforts.

## Introduction

Telehealth provides a possible solution to the problem of rural health disparities in the United States.^1–7^ However, many telehealth solutions require access to consistent, reliable, and affordable broadband of sufficient speed and quality.^8^ Broadband has historically been defined by the Federal Communications Commission (FCC) as download speeds of at least 25 megabits per second (Mbps) and upload speeds of at least 3 Mbps (25/3 Mbps),^9,10^ although this benchmark has recently increased to 100/20 Mbps.^11^ Unequal broadband access in rural and other underserved areas of the United States has become a critical issue for health equity,^12–15^ and although broadband usage through mobile devices and networks has increased,^16,17^ rural and other underserved communities still lack sufficient speeds to participate in many online activities, including telehealth.^18–21^ Previous research has found that better broadband access is associated with increased telehealth utilization.^22^ Additionally, isolated rural medically underserved areas (MUAs) demonstrated less improvement in broadband access than other areas.^23^ However, a challenge that persists in studying the relationship between broadband, telehealth, and health care access is a lack of high-quality broadband data.

To address the digital divide, broadband policies and interventions must first assess broadband data sources to identify where consumer broadband access is lacking. This is particularly critical in light of the historic Broadband Equity and Access Deployment (BEAD) Program,^24^ which requires states to prioritize broadband investment based on broadband access data.^25^ Historically, measuring broadband access has been a difficult challenge to overcome.^26,27^ One of the most widely used, publicly available sources of consumer broadband access comes from the FCC’s Form 477. This form is completed twice per year by internet service providers and reports the fastest advertised download and upload speeds greater than 200 kilobits per second (Kbps) offered to consumers within a census block.^28,29^ However, these data obscure variation within census blocks, provide no direct observations, obscure temporal variation, and may not match speeds consumers are receiving.^30–32^ This process may be especially detrimental to rural areas, where census blocks cover larger areas and unserved homes may be far from existing broadband infrastructure. Estimates of the number of Americans who lack broadband access range from 14.5 million to 42 million, with rural consumers more likely to lack access.^33,34^ Thus, direct measurement of internet speeds could improve our understanding of which areas need additional broadband investment before they are telehealth-ready.

Some of the most promising data sources of consumer broadband access come from user-generated speed tests.^35^ Two of the largest sources of consumer-generated internet speed test data are Measurement Labs (M-Lab)^36^ and Ookla for Good (Ookla).^37^ Both services allow users to assess internet connection speeds through a web browser or dedicated smartphone application and assess the quality of their connection with metrics such as download speed, upload speed, and latency. However, the testing protocols utilized by these two sources differ in a number of technical ways, such as the location of the servers receiving data sent by users and simultaneous (Ookla) or single (M-Lab) streams of data, among others.^38,39^ Additionally, in terms of information available to the public, M-Lab data are temporally precise, with individual speed test data available with microsecond precision,^40^ but spatially less precise, with geographic information available only through an imprecise IP-address geolocation lookup.^41,42^ Publicly available Ookla internet speed test data are aggregated across three-month periods for a series of squares measuring 610.8 m^2^ across the globe using Global Positioning System (GPS) technology:^43^ spatially more precise but temporally less precise than M-Lab data. Both data sets have important limitations but may still be a resource to assess broadband access at the consumer/patient level. Previous studies using these data sets have found that rural areas are more likely to have slower download speeds, but these studies also identify a lack of reliable data from some study areas as a limitation of these data sets.^44,45^ To the best of our knowledge, no study has examined the relationship between rurality and other sociodemographic characteristics with a population-standardized number of user-generated speed tests in these data sets. As these data sets are already being used to assess broadband access and quality, it is important to understand the limitations of these data sets, such as what factors contribute to inclusion in the data sets themselves. For example, if the number of user-generated speed tests per 1,000 residents is lower in rural relative to urban counties, this outcome raises an issue about the reliability of the connection speeds and latencies measured for those areas. Many rural areas may be affected by the digital divide,^46^ and lack of accurate representation in data sets used by researchers and policymakers to determine broadband access and investment is a serious concern. Thus, in the current study, we fit several models using the number of speed tests from M-Lab and Ookla in rural and urban U.S. counties between January 2020 and December 2021 per 1,000 residents as the outcome variable of interest and county-level rurality as the main independent variable of interest, and we included several additional sociodemographic covariates based on a review of the broadband access literature. We hypothesized the tests per 1,000 residents among rural consumers would be lower for rural residents.

## Methods

### Data sources

We included M-Lab, Ookla fixed, and Ookla mobile speed test data in our analyses, as well as data from the Area Health Resource File (AHRF), and American Community Survey (ACS) for county-level covariates. M-Lab data were accessed using Google Cloud Platform’s (GCPs) Public BigQuery^47^ using download (‘measurement-lab.ndt.unified_downloads’) and upload speed (‘measurement-lab.ndt.unified_uploads’) based on the unified view of the Network Diagnostic Tool (NDT) performance measure. The NDT is a series of open-source protocols to evaluate a user’s internet connection that has been updated over time.^48^ When a user/client makes a request, the NDT “executes a ten-second download from an M-Lab server to the client, and an upload from the client to that server;…it reports and records the speed of these transfers, as well as a number of other measures, such as the minimum and final round trip time (RTT) between client and server.”^49^ M-Lab NDT data were joined to U.S. county boundary information via ‘bigquery-public-data.geo_us_boundaries.counties’.

Ookla speed test data were accessed via Ookla’s open data platform.^43^ Two separate Ookla data sets were downloaded: fixed and mobile. Ookla fixed data sets contained speed tests conducted over wired or wireless fixed connections, such as satellite, digital subscriber lines (DSL), cable, and fiber. Ookla mobile data sets contained speed tests conducted over cellular data. Both Ookla data sets contain the number of speed tests conducted within any 610.8 m^2^ tiles with at least one recorded test during each quarter. Ookla data were merged with county shape files downloaded from www.census.gov.^50^

AHRF 2020–2021 data were obtained from www.HRSA.gov.^51^ ACS five-year estimates from 2015–2019 were obtained from www.census.gov.^52^

### Study design

We conducted a longitudinal panel study using national M-Lab and Ookla fixed/mobile data aggregated at the U.S. county level, by quarters across 2020 and 2021. Quarters were chosen as the temporal level of analysis because they are the smallest analyzable units available in the Ookla data set. Counties were chosen as the geographic level of analysis because they are the smallest analyzable unit available in the M-Lab data set.^42^

### Study outcome measure

We used the number of speed tests conducted per 1,000 residents at the county level as the outcome variable in our models. The data for both fixed and mobile Ookla data were adjusted for tiles crossing county lines by dividing the total number of speed tests performed in that tile by the total number of counties represented by that tile (tiles overlapped a maximum of four counties; 4.2% of all tiles).

### Main independent variable

To examine the distribution of the number of speed tests conducted per 1,000 residents in rural/urban areas, publicly available Rural-Urban Continuum Code (RUCC) data were downloaded from the United States Department of Agriculture’s Economic Research Service website and introduced as categorical independent variables.^53^ Analyses were restricted to all counties and county-equivalents (henceforth counties) in the 50 U.S. states and the District of Columbia, yielding 3,142 counties.

### Covariates

County-level covariates were identified based on a review of previous literature examining predictors of consumer broadband access, particularly Reddick et al.^15,54–57^ and included from AHRF and ACS data, including percentage of county residents identifying as non-White/non-Hispanic, average household size, percentage of residents over the age of 25 years with a high school diploma, unemployment rate for residents over 16% of residents in poverty, percentage of residents over 65% of female residents, percentage of non-English-speaking households, and percentage of county residents reporting owning or using a computer with household broadband. Covariates were transformed through mean-centering for ease of coefficient interpretation.

### Statistical analyses

The number of speed tests conducted per 1,000 residents was fit to negative binomial distribution regression models, with standard errors clustered at the state level. Coefficients, incidence rate ratios (IRRs), confidence intervals (CIs), and *p* values are reported for all models. Analyses were performed using R version 4.1.0 (R Foundation for Statistical Computing, Vienna, Austria) and RStudio version 2023.6.0.421 (RStudio, PBC, Boston, MA) with the ‘fixest’ and ‘tidyverse’ packages installed.^58–61^ A two-sided significance level of 0.05 was set *a priori*. This study was reviewed by the [Institutional Review Board at the University of Arkansas for Medical Sciences and determined nonhuman subjects research (#262566).

## Results

Across 3,142 U.S. counties/county equivalents over 2020 and 2021, there were a total of 588,010,514 M-Lab speed tests, 185,914,683 fixed Ookla speed tests, and 22,759,514 mobile Ookla speed tests. Table 1 includes the total number of speed tests for each data source across all quarters in 2020 and 2021, and Table 2 includes the mean, standard deviation, and median number of speed tests per 1,000 county residents by RUCC for each data source. The results of the negative binomial regression fitting the M-Lab data (Table 3) show fewer speed tests conducted per 1,000 residents in all but three RUCCs relative to RUCC 1 (RUCC 2: IRR = 0.81, *p* = 0.002; RUCC 3: IRR = 0.73, *p* < 0.001; RUCC 6: IRR = 0.69, *p* < 0.001; RUCC 7: IRR = 0.74, *p* = 0.002; RUCC 9: IRR = 0.57, *p* < 0.001). No other RUCC comparisons reached significance. The number of speed tests conducted per 1,000 residents was also larger in all quarters relative to 2020 Q1 (2020 Q2: IRR = 1.25, *p* < 0.001; 2020 Q3: IRR = 1.30, *p* < 0.001; 2020 Q4: IRR = 1.52, *p* < 0.001; 2021 Q1: IRR = 1.49, *p* < 0.001; 2021 Q2: IRR = 1.42, *p* < 0.001; 2021 Q3: IRR = 1.41, *p* < 0.001; 2021 Q4: IRR = 1.40, *p* < 0.001).

**Table 1.** Number of Counties with Total Numbers of Tests by Data Source Across All Quarters in 2020–2021

			Ookla			
	M-Lab		Fixed		Mobile	
	*n*	%	*n*	%	*n*	%
Under 100 Tests	22	0.7%	44	1.4%	151	4.8%
More than 100, Under 1,000 Tests	150	4.8%	219	7.0%	1,038	33.0%
More than 1,000, Under 10,000 Tests	963	30.6%	1,284	40.9%	1,537	48.9%
More than 10,000, Under 100,000 Tests	1,397	44.5%	1,228	39.1%	386	12.3%
More than 100,000, Under 1,000,000 Tests	496	15.8%	341	10.9%	30	1.0%
More than 1,000,000, Under 10,000,000 Tests	110	3.5%	26	0.8%	0	0.0%
More than 10,000,000 Tests	4	0.1%	0	0.0%	0	0.0%

**Table 2.** Total Speed Tests per RUCC and Mean, SD, and Median Number of Speed Tests per 1,000 Residents by Nine RUCC Categories for M-Lab, Ookla Fixed, and Ookla Mobile in 2020–2021

		M-Lab				Ookla							
						Fixed				Mobile			
		Overall	Tests per 1,000 residents			Overall	Tests per 1,000 residents			Overall	Tests per 1,000 residents		
Rural-Urban Continuum Code (RUCC)		Total Tests	Mean	SD	Median	Total Tests	Mean	SD	Median	Total Tests	Mean	SD	Median
1	Counties in metro areas of 1 million population or more	395,167,880	195.6	404.7	139.2	114,080,521	68.4	32.2	63.6	13,400,848	8.7	4	8
2	Counties in metro areas of 250,000 to 1 million population	94,405,405	133.2	157.5	110.7	37,516,270	61	25.5	57.3	4,527,961	8.2	4.3	7.3
3	Counties in metro areas of fewer than 250,000 population	32,158,587	115.5	137.8	99.1	14,499,378	55.7	25.4	52.2	1,911,296	7.8	4.8	6.9
4	Urban population of 20,000 or more, adjacent to a metro area	39,006,437	369.9	3,533.3	101.8	6,121,767	55.9	25.4	50.9	861,853	7.9	3.9	6.9
5	Urban population of 20,000 or more, not adjacent to a metro area	5,804,977	133	131.1	101.9	2,307,194	54.7	24.4	49.6	297,791	7.3	4.3	6.4
6	Urban population of 2,500 to 19,999, adjacent to a metro area	11,202,313	93.4	172.3	74.2	5,876,727	47.8	27.8	41.9	962,711	8.2	6.9	6.8
7	Urban population of 2,500 to 19,999, not adjacent to a metro area	6,892,110	104	105.7	83.5	3,593,959	51.7	34.3	44.5	487,539	7.8	6.4	6.1
8	Completely rural or less than 2,500 urban population, adjacent to a metro area	1,797,155	100.5	658.2	55.2	912,606	50.9	52.5	40	152,583	9.1	11.7	6.3
9	Completely rural or less than 2,500 urban population, not adjacent to a metro area	1,575,650	72.4	90.9	46.9	1,006,261	45.8	60.4	34.2	156,932	9.4	26.1	4.9

**Table 3.** Results of the M-Lab Number of Speed Tests per 1,000 Residents by County Negative Binomial Regression Results (*n* = 25,128 County-Quarter Observations)

	Coefficient	IRR	95% CI	*p*-value
(Intercept)	4.06	58.18	[2.16, 5.96]	<0.001
Rural-Urban Commuting Code (RUCC)				
Counties in metro areas of 1 million population or more (1)	(ref)	—	—	—
Counties in metro areas of 250,000 to 1 million population (2)	−0.21	0.81	[−0.35, −0.08]	0.002
Counties in metro areas of fewer than 250,000 population (3)	−0.32	0.73	[−0.48, −0.16]	<0.001
Urban population of 20,000 or more, adjacent to a metro area (4)	0.86	2.36	[−0.32, 2.04]	0.15
Urban population of 20,000 or more, not adjacent to a metro area (5)	−0.16	0.85	[−0.38, 0.06]	0.16
Urban population of 2,500 to 19,999, adjacent to a metro area (6)	−0.37	0.69	[−0.57, −0.16]	<0.001
Urban population of 2,500 to 19,999, not adjacent to a metro area (7)	−0.3	0.74	[−0.5, −0.11]	0.002
Completely rural or less than 2,500 urban population, adjacent to a metro area (8)	−0.23	0.8	[−0.63, 0.18]	0.28
Completely rural or less than 2,500 urban population, not adjacent to a metro area (9)	−0.56	0.57	[−0.82, −0.31]	<0.001
Year/Quarter				
2020 Q1	(ref)	—	—	—
2020 Q2	0.24	1.28	[0.2, 0.29]	<0.001
2020 Q3	0.33	1.39	[0.26, 0.39]	<0.001
2020 Q4	0.5	1.64	[0.44, 0.55]	<0.001
2021 Q1	0.48	1.61	[0.42, 0.54]	<0.001
2021 Q2	0.54	1.71	[0.41, 0.66]	<0.001
2021 Q3	0.48	1.62	[0.36, 0.6]	<0.001
2021 Q4	0.35	1.41	[0.29, 0.4]	<0.001
Percent non-White/non-Hispanic	0	1	[0, 0.01]	0.29
Average household size	−0.68	0.5	[−1.03, −0.34]	<0.001
Unemployment rate 16+	−0.01	0.99	[−0.03, 0.02]	0.56
Percent persons in poverty	−0.03	0.97	[−0.04, −0.01]	0.002
Percent population 65+	−0.02	0.98	[−0.02, −0.01]	<0.001
Percent female	0.03	1.03	[0.01, 0.05]	0.001
Percent non-English-speaking households	0.05	1.05	[0.03, 0.07]	<0.001
Percent has a computer and broadband connection	0.02	1.02	[0, 0.03]	0.02

Average household size (IRR = 0.68, *p* = 0.002), percentage of persons in poverty (IRR = 0.87, *p* < 0.001), and percentage of the population over the age of 65 years (IRR = 0.87, *p* = 0.007) were negatively associated with the number of M-Lab speed tests conducted per 1,000 residents. Additionally, the percentage of female residents (IRR = 1.03, *p* = 0.001), the percentage of non-English-speaking households (IRR = 1.05, *p* < 0.001), and the percentage of households with a computer and a broadband connection (IRR = 1.02, *p* = 0.02) were all positively associated with the number of M-Lab speed tests conducted per 1,000 residents. No other covariates emerged as significant in the model.

In a sensitivity analysis removing the highest and lowest 1% of county-quarter observations from the fitted model, we observed similar results (see Appendix 1).

The results of the negative binomial regression fitting the Ookla fixed data (Table 4) showed fewer speed tests conducted per 1,000 residents in RUCC 6 counties (IRR = 0.85, *p* = 0.003) and RUCC 9 counties (IRR = 0.79, *p* = 0.01) relative to RUCC 1 counties. No other county comparisons emerged as significant. There was a significant increase in the number of Ookla fixed speed tests conducted per 1,000 residents from 2020 Q1 to 2020 Q2 (IRR = 1.13, *p* < 0.001) and significant decreases between 2020 Q1 and all remaining quarters (2020 Q4: IRR = 0.72, *p* < 0.001; 2021 Q1: IRR = 0.80, *p* < 0.001; 2021 Q2: IRR = 0.77, *p* < 0.001; 2021 Q3: IRR = 0.86, *p* < 0.001; 2021 Q4: IRR = 0.80, *p* < 0.001).

**Table 4.** Ookla Number of Fixed Speed Tests per 1,000 Residents by County Negative Binomial Regression Results (*n* = 25,128 County-Quarter Observations)

	Coefficient	IRR	95% CI	*p*-value
(Intercept)	2.05	7.75	[0.3, 3.8]	0.02
Rural-Urban Commuting Code (RUCC)				
Counties in metro areas of 1 million population or more (1)	(ref)	—	—	—
Counties in metro areas of 250,000 to 1 million population (2)	−0.03	0.97	[−0.11, 0.05]	0.47
Counties in metro areas of fewer than 250,000 population (3)	−0.08	0.92	[−0.16, 0]	0.05
Urban population of 20,000 or more, adjacent to a metro area (4)	−0.09	0.92	[−0.19, 0.02]	0.1
Urban population of 20,000 or more, not adjacent to a metro area (5)	−0.06	0.95	[−0.18, 0.07]	0.4
Urban population of 2,500 to 19,999, adjacent to a metro area (6)	−0.16	0.85	[−0.27, −0.05]	0.003
Urban population of 2,500 to 19,999, not adjacent to a metro area (7)	−0.11	0.9	[−0.23, 0.01]	0.08
Completely rural or less than 2,500 urban population, adjacent to a metro area (8)	−0.11	0.9	[−0.27, 0.06]	0.21
Completely rural or less than 2,500 urban population, not adjacent to a metro area (9)	−0.23	0.79	[−0.41, −0.05]	0.01
Year/Quarter				
2020 Q1	(ref)	—	—	—
2020 Q2	0.12	1.13	[0.1, 0.15]	<0.001
2020 Q3	0.01	1.01	[−0.03, 0.05]	0.73
2020 Q4	−0.33	0.72	[−0.36, −0.3]	<0.001
2021 Q1	−0.22	0.8	[−0.25, −0.2]	<0.001
2021 Q2	−0.26	0.77	[−0.3, −0.23]	<0.001
2021 Q3	−0.15	0.86	[−0.2, −0.1]	<0.001
2021 Q4	−0.23	0.8	[−0.25, −0.2]	<0.001
Percent non-White/non-Hispanic	0	1	[−0.01, 0]	0.054
Average household size	−0.01	0.99	[−0.28, 0.26]	0.95
Unemployment rate 16+	0.04	1.04	[0.01, 0.06]	0.002
Percent persons in poverty	−0.02	0.98	[−0.03, 0]	0.007
Percent population 65+	0.01	1.01	[0, 0.02]	0.007
Percent female	0.01	1.01	[0, 0.03]	0.17
Percent non-English-speaking households	0.01	1.01	[0, 0.02]	0.03
Percent has a computer and broadband connection	0.02	1.02	[0.01, 0.02]	<0.001

We observed significant positive associations between the number of Ookla fixed speed tests conducted per 1,000 residents and the unemployment rate (IRR = 1.04, *p* = 0.002), the percentage of the population over the age of 65 years (IRR = 1.01, *p* = 0.007), the percentage of non-English-speaking households (IRR = 1.01, *p* = 0.03), and the percentage of households with a computer and broadband connection (IRR = 1.02, *p* < 0.001). We found a significant negative association between the number of Ookla fixed speed tests conducted per 1,000 residents and the percentage of residents in poverty (IRR = 0.98, *p* = 0.007). No other covariates reached statistical significance.

A sensitivity analysis trimming the highest and lowest 1% of all county-quarter observations of Ookla fixed speed tests conducted per 1,000 residents found similar results (see Appendix 2). However, in this analysis, RUCC 3, 7, and 8 also had fewer Ookla fixed speed tests conducted per 1,000 residents relative to RUCC 1 counties (RUCC 3: IRR = 0.91, *p* = 0.02; RUCC 7: IRR = 0.88, *p* = 0.03; RUCC 8: IRR = 0.85, *p* = 0.02). Additionally, the percentage of non-White/non-Hispanic residents was negatively associated with the number of speed tests conducted per 1,000 residents (IRR = 0.996, *p* = 0.03), and the percentage of female residents was positively associated (IRR = 1.02, *p* = 0.004).

The results of the negative binomial regression fitting the Ookla mobile data (Table 5) showed no differences in the number of speed tests conducted per 1,000 residents among any of the RUCC levels of rurality (all *p* values >0.05). The number of Ookla mobile speed tests conducted per 1,000 residents increased in each quarter relative to 2020 Q1 (2020 Q2: IRR = 1.12, *p* < 0.001; 2020 Q3: IRR = 1.31, *p* < 0.001; 2020 Q4: IRR = 1.26, *p* < 0.001; 2021 Q1: IRR = 1.18, *p* < 0.001; 2021 Q2: IRR = 1.27, *p* < 0.001; 2021 Q3: IRR = 1.33, *p* < 0.001; 2021 Q4: IRR = 1.20, *p* < 0.001). A negative association was observed between the number of Ookla mobile speed tests conducted per 1,000 residents, the percentage of persons in poverty (IRR = 0.98, *p* = 0.02), and the percentage of female residents (IRR = 0.95, *p* < 0.001). The percentage of non-English-speaking households was positively associated with the number of Ookla mobile speed tests conducted per 1,000 residents (IRR = 1.03, *p* = 0.001). No other covariates reached statistical significance.

**Table 5.** Ookla Number of Mobile Speed Tests per 1,000 Residents by County Negative Binomial Regression Results (*n* = 25,128 County-Quarter Observations)

	Coefficient	IRR	95% CI	*p*-value
(Intercept)	4.66	105.51	[1.71, 7.6]	0.002
Rural-Urban Commuting Code (RUCC)				
Counties in metro areas of 1 million population or more (1)	(ref)	—	—	—
Counties in metro areas of 250,000 to 1 million population (2)	−0.03	0.97	[−0.11, 0.05]	0.44
Counties in metro areas of fewer than 250,000 population (3)	−0.07	0.94	[−0.16, 0.03]	0.19
Urban population of 20,000 or more, adjacent to a metro area (4)	−0.07	0.93	[−0.18, 0.04]	0.23
Urban population of 20,000 or more, not adjacent to a metro area (5)	−0.13	0.88	[−0.27, 0.01]	0.07
Urban population of 2,500 to 19,999, adjacent to a metro area (6)	−0.06	0.94	[−0.16, 0.05]	0.28
Urban population of 2,500 to 19,999, not adjacent to a metro area (7)	−0.14	0.87	[−0.29, 0.01]	0.06
Completely rural or less than 2,500 urban population, adjacent to a metro area (8)	−0.01	0.99	[−0.22, 0.2]	0.93
Completely rural or less than 2,500 urban population, not adjacent to a metro area (9)	−0.06	0.94	[−0.32, 0.21]	0.66
Year/Quarter				
2020 Q1	(ref)	—	—	—
2020 Q2	0.11	1.12	[0.06, 0.16]	<0.001
2020 Q3	0.27	1.31	[0.18, 0.36]	<0.001
2020 Q4	0.23	1.26	[0.17, 0.28]	<0.001
2021 Q1	0.17	1.18	[0.13, 0.21]	<0.001
2021 Q2	0.24	1.27	[0.16, 0.32]	<0.001
2021 Q3	0.28	1.33	[0.2, 0.37]	<0.001
2021 Q4	0.18	1.2	[0.14, 0.22]	<0.001
Percent non-White/non-Hispanic	0	1	[0, 0]	0.65
Average household size	−0.08	0.92	[−0.61, 0.45]	0.76
Unemployment rate 16+	0.01	1.01	[−0.02, 0.04]	0.4
Percent persons in poverty	−0.02	0.98	[−0.04, 0]	0.02
Percent population 65+	0.02	1.02	[0, 0.04]	0.1
Percent female	−0.05	0.95	[−0.07, −0.03]	<0.001
Percent non-English-speaking households	0.03	1.03	[0.01, 0.05]	0.001
Percent has a computer and broadband connection	−0.001	0.999	[−0.01, 0.01]	0.74

Similar results were observed in a sensitivity analysis removing the highest and lowest 1% of all Ookla mobile speed tests conducted per 1,000 residents (see Appendix 3). However, in this analysis, RUCC 7 counties had fewer Ookla mobile speed tests conducted per 1,000 residents relative to RUCC 1 counties (IRR = 0.87, *p* = 0.04). Additionally, the percentage of county residents over the age of 65 years was positively associated with the number of Ookla mobile speed tests conducted per 1,000 residents (IRR = 1.03, *p* < 0.001).

## Discussion

Using publicly available data sources of the number of internet speed tests conducted per 1,000 residents during 2020 and 2021, we found that county-level rurality was generally associated with fewer speed tests conducted per 1,000 residents in analyses of fixed data, but not in mobile data. However, these effects were heavily influenced by outliers. All three data sources contained fewer than 100 speed tests across all quarters for some counties. Additionally, some counties were overrepresented in the data. For example, the number of M-Lab speed tests conducted per 1,000 residents for Reno County, Kansas (RUCC 4), was above 27,000 in every quarter (RUCC 4 median number of speed tests: 101.8). This finding underscores how these data may be biased in unexpected ways. In sensitivity analyses removing the highest and lowest 1% of observations from both fixed data sets, all rural counties with fewer than 2,500 residents—regardless of adjacency to a metro area—were negatively associated with the number of speed tests conducted per 1,000 residents. As has been found in prior studies,^62–68^ internet speeds and thus access to consumer broadband are lower in rural areas than in urban areas. Our finding that rural counties are poorly represented in some sources of speed tests controlling for county population raises concern that these measures of consumer broadband access may not be valid for many rural areas where telehealth services and increased health access are most needed. This finding has important implications for future research and policy, as these data may be used to evaluate where broadband access and investment are most needed, but these may not accurately reflect the state of broadband in all areas. Our study highlights the importance of evaluating data sources when making broadband-related policy decisions, as well as the need for additional data collection in rural areas of the United States.

We had hypothesized that the number of speed tests conducted per 1,000 residents would increase over time relative to the first quarter of 2020, as this period of the COVID-19 public health emergency (PHE) included lockdowns, remote education, and shifts to remote work in many sectors,^69^ which has been found to be associated with increased internet traffic.^70^ We did observe an increase in the number of speed tests conducted per 1,000 residents for both M-Lab and Ookla mobile data sets for all quarters of 2020 and 2021 relative to the first quarter of 2020, regardless of outliers. The results from the Ookla fixed data set, however, demonstrated increases in the number of speed tests conducted per 1,000 residents only for the second quarter of 2020, no change in the third quarter of 2020, and then decreases in the number of speed tests conducted per 1,000 residents in the remaining quarter of 2020 and all quarters of 2021, regardless of outliers. These findings highlight the challenge of using a single data source to assess broadband access, as the effect of the COVID-19 PHE on the number of speed tests conducted per 1,000 residents varies depending on the specific data source, even among data sources exclusively measuring user-generated speed tests.

In the Ookla mobile data, we did not find significant associations between county-level rurality and the number of speed tests conducted per 1,000 residents. This result is consistent with the finding that the digital divide between rural and urban communities is shrinking for mobile connections, unlike the divide in fixed connections.^13,56^ Ownership of smartphones is increasing in rural areas faster than ownership of tablets or personal computers.^71^ However, mobile broadband currently does not offer the same benefits as a fixed connection, as mobile connections are typically not as reliable, consistent, or fast as fixed connections, making them less preferred for video calls, such as a telehealth appointments.^72^ Future work is needed to further examine the landscape of the digital divide for different types of broadband connections.

Only the county-level percentage of persons in poverty was a consistent negative predictor of the number of speed tests conducted per 1,000 residents across the M-Lab, Ookla fixed, and Ookla mobile data sets, although the IRR was fairly low in all models. The percentage of non-English-speaking households was the only covariate with a consistent positive association with the number of speed tests per 1,000 residents in all three models. Having a computer and broadband connection was positively associated with the number of speed tests per 1,000 residents in both fixed models but not in the Ookla mobile model. This result underscores the importance of considering network connection type when examining consumer broadband access and the digital divide, particularly for rural/urban comparisons.^13,56^ The lack of other consistent predictors across all models, including sensitivity models with outliers removed, suggests that these data sources represent consumer broadband access among slightly different populations.

### Limitations

The results of our study must be interpreted carefully. First, to compare M-Lab and Ookla data, we mapped speed test locations to counties because of the lack of more granular geographical information available in the M-Lab data. M-Lab data less reliably identify a speed test’s county origin, and this process may be less accurate for rural areas than urban areas.^73^ Additionally, many counties are heterogeneous in terms of the mix of rural and urban areas, which may obscure important geographical variations within counties. A future examination of Ookla data alone mapped to smaller geographical regions may shed light on where broadband information is most lacking more precisely.

We also found differences in factors associated with the number of internet speed tests conducted in our three models, suggesting some or all of these data sets may be biased. Both M-Lab and Ookla must contend with issues of data quality and sampling, such as individual users conducting many tests from the same device,^49^ which could bias aggregated measures such as download/upload speed. M-Lab and Ookla utilize different strategies to address these issues,^48,74^ and it is possible that our results are influenced by different data cleaning processes conducted.

Finally, we did not examine other known barriers to consumer broadband access in this study, such as high cost, data caps, network outages, and temporal variation in connection speed and quality,^75^ which may also disproportionately affect underserved populations such as rural residents. Additional data sources and research are needed to examine how these barriers may be inequitably distributed across broadband consumers.

## Conclusion

With federal and state interest in broadband infrastructure and narrowing the digital divide, the need for consumer-level data on broadband access is critical. However, some of the communities in the greatest need of broadband infrastructure investment (i.e., rural) may have too few observations for reliable estimates of broadband access. Our study examined the number of M-Lab and Ookla speed tests conducted per 1,000 county residents and found that those data may be based on too few data points for valid estimates. Although high-quality data are critical to understanding the state of broadband and the digital divide for rural areas, many of these data sources may be biased due to the nature of how they are used by consumers and/or shared with the public. Researchers invested in digital health solutions for patients in rural areas need to be aware of how much is yet unknown about the state of consumer broadband in these areas and work to fill the research gap to identify where patients can access broadband and make use of telehealth interventions.

##  

## Abbreviations Used

AHRF: Area Health Resource File
BEAD: Broadband Equity and Access Deployment
DSL: digital subscriber lines
FCC: Federal Communications Commission
GCPs: Google Cloud Platform’s
GPS: Global Positioning System
IRR: incidence rate ratios
Mbps: Megabits Per Second
M-Lab: Measurement Labs
MUAs: Medically Underserved Areas
NDT: Network Diagnostic Tool
RTT: Round Trip Time
RUCC: Rural-Urban Continuum Code
U.S.: United State

## Appendix 1

**Table A1.** Results of a Sensitivity Analysis After Removing the Highest and Lowest 1% of Observations and Fitting the Data to a Negative Binomial Regression of M-Lab Number of Speed Tests per 1,000 Residents by County (*n* = 24,320 County-Quarter Observations)

	Coefficient	IRR	95% CI	*p* value
(Intercept)	2.75	15.62	[1.52, 3.97]	<0.001
Rural-Urban Commuting Code (RUCC)				
Counties in metro areas of 1 million population or more (1)	(ref)	—	—	—
Counties in metro areas of 250,000 to 1 million population (2)	−0.08	0.92	[−0.16, −0.01]	0.03
Counties in metro areas of fewer than 250,000 population (3)	−0.14	0.87	[−0.23, −0.05]	0.002
Urban population of 20,000 or more, adjacent to a metro area (4)	−0.05	0.95	[−0.13, 0.03]	0.18
Urban population of 20,000 or more, not adjacent to a metro area (5)	−0.04	0.96	[−0.13, 0.05]	0.4
Urban population of 2,500 to 19,999, adjacent to a metro area (6)	−0.23	0.8	[−0.31, −0.14]	<0.001
Urban population of 2,500 to 19,999, not adjacent to a metro area (7)	−0.14	0.87	[−0.24, −0.04]	0.006
Completely rural or less than 2,500 urban population, adjacent to a metro area (8)	−0.31	0.73	[−0.45, −0.17]	<0.001
Completely rural or less than 2,500 urban population, not adjacent to a metro area (9)	−0.43	0.65	[−0.59, −0.28]	<0.001
Year/Quarter				
2020 Q1	(ref)	—	—	—
2020 Q2	0.22	1.25	[0.2, 0.25]	<0.001
2020 Q3	0.26	1.3	[0.23, 0.3]	<0.001
2020 Q4	0.42	1.52	[0.38, 0.45]	<0.001
2021 Q1	0.4	1.49	[0.36, 0.44]	<0.001
2021 Q2	0.35	1.42	[0.32, 0.39]	<0.001
2021 Q3	0.35	1.41	[0.31, 0.38]	<0.001
2021 Q4	0.33	1.4	[0.3, 0.37]	<0.001
Percent non-White/non-Hispanic	0	1	[0, 0]	0.6
Average household size	−0.38	0.68	[−0.63, −0.14]	0.002
Unemployment rate 16+	0.03	1.03	[0.01, 0.04]	<0.001
Percent persons in poverty	−0.02	0.98	[−0.03, −0.01]	<0.001
Percent population 65+	−0.01	0.99	[−0.01, 0]	0.01
Percent female	0.02	1.02	[0.01, 0.04]	0.003
Percent non-English-speaking households	0.02	1.02	[0, 0.04]	0.02
Percent has a computer and broadband connection	0.02	1.02	[0.01, 0.03]	<0.001

## Appendix 2

**Table A2.** Results of a Sensitivity Analysis After Removing the Highest and Lowest 1% of Observations and Fitting the Data to a Negative Binomial Regression of Ookla Number of Fixed Speed Tests per 1,000 Residents by County (*n* = 24,893 County-Quarter Observations)

	Coefficient	IRR	95% CI	*p*-value
(Intercept)	1.77	5.88	[0.43, 3.11]	0.009
Rural-Urban Commuting Code (RUCC)				
Counties in metro areas of 1 million population or more (1)	(ref)	—	—	—
Counties in metro areas of 250,000 to 1 million population (2)	−0.04	0.96	[−0.12, 0.05]	0.37
Counties in metro areas of fewer than 250,000 population (3)	−0.09	0.91	[−0.17, −0.01]	0.02
Urban population of 20,000 or more, adjacent to a metro area (4)	−0.1	0.91	[−0.2, 0]	0.052
Urban population of 20,000 or more, not adjacent to a metro area (5)	−0.07	0.94	[−0.19, 0.06]	0.29
Urban population of 2,500 to 19,999, adjacent to a metro area (6)	−0.18	0.84	[−0.28, −0.08]	<0.001
Urban population of 2,500 to 19,999, not adjacent to a metro area (7)	−0.13	0.88	[−0.24, −0.01]	0.03
Completely rural or less than 2,500 urban population, adjacent to a metro area (8)	−0.16	0.85	[−0.3, −0.02]	0.02
Completely rural or less than 2,500 urban population, not adjacent to a metro area (9)	−0.26	0.77	[−0.42, −0.11]	<0.001
Year/Quarter				
2020 Q1	(ref)	—	—	—
2020 Q2	0.12	1.13	[0.1, 0.14]	<0.001
2020 Q3	0	1	[−0.04, 0.03]	0.86
2020 Q4	−0.32	0.72	[−0.35, −0.3]	<0.001
2021 Q1	−0.21	0.81	[−0.24, −0.19]	<0.001
2021 Q2	−0.27	0.76	[−0.3, −0.23]	<0.001
2021 Q3	−0.16	0.85	[−0.21, −0.12]	<0.001
2021 Q4	−0.22	0.8	[−0.25, −0.19]	<0.001
Percent non-White/non-Hispanic	0	1	[−0.01, 0]	0.03
Average household size	0.05	1.06	[−0.19, 0.3]	0.65
Unemployment rate 16+	0.04	1.04	[0.01, 0.06]	0.001
Percent persons in poverty	−0.02	0.98	[−0.03, 0]	0.01
Percent population 65+	0.01	1.01	[0.01, 0.02]	<0.001
Percent female	0.02	1.02	[0.01, 0.03]	0.004
Percent non-English-speaking households	0.01	1.01	[0, 0.02]	0.02
Percent has a computer and broadband connection	0.01	1.01	[0.01, 0.02]	<0.001

## Appendix 3.

**Table A3.** Results of a Sensitivity Analysis After Removing the Highest and Lowest 1% of Observations and Fitting the Data to a Negative Binomial Regression of Ookla Number of Mobile Speed Tests per 1,000 Residents by County (*n* = 24,677 County-Quarter Observations)

	Coefficient	IRR	95% CI	*p*-value
(Intercept)	3.4	30.08	[1.57, 5.24]	<0.001
Rural-Urban Commuting Code (RUCC)	(ref)	—	—	—
Counties in metro areas of 1 million population or more (1)	(ref)	—	—	—
Counties in metro areas of 250,000 to 1 million population (2)	−0.05	0.95	[−0.12, 0.03]	0.21
Counties in metro areas of fewer than 250,000 population (3)	−0.08	0.93	[−0.17, 0.02]	0.11
Urban population of 20,000 or more, adjacent to a metro area (4)	−0.09	0.92	[−0.19, 0.02]	0.1
Urban population of 20,000 or more, not adjacent to a metro area (5)	−0.13	0.88	[−0.26, 0]	0.052
Urban population of 2,500 to 19,999, adjacent to a metro area (6)	−0.09	0.92	[−0.18, 0.01]	0.08
Urban population of 2,500 to 19,999, not adjacent to a metro area (7)	−0.14	0.87	[−0.28, 0]	0.04
Completely rural or less than 2,500 urban population, adjacent to a metro area (8)	−0.02	0.98	[−0.22, 0.18]	0.86
Completely rural or less than 2,500 urban population, not adjacent to a metro area (9)	−0.06	0.94	[−0.24, 0.12]	0.5
Year/Quarter	(ref)	—	—	—
2020 Q1	(ref)	—	—	—
2020 Q2	0.13	1.14	[0.1, 0.16]	<0.001
2020 Q3	0.29	1.33	[0.22, 0.35]	<0.001
2020 Q4	0.23	1.26	[0.19, 0.27]	<0.001
2021 Q1	0.17	1.19	[0.14, 0.21]	<0.001
2021 Q2	0.24	1.27	[0.17, 0.31]	<0.001
2021 Q3	0.28	1.32	[0.2, 0.36]	<0.001
2021 Q4	0.18	1.19	[0.14, 0.22]	<0.001
Percent non-White/non-Hispanic	0	1	[0, 0]	0.58
Average household size	0.17	1.18	[−0.21, 0.55]	0.38
Unemployment rate 16+	0.02	1.02	[−0.01, 0.04]	0.2
Percent persons in poverty	−0.02	0.98	[−0.03, 0]	0.02
Percent population 65+	0.03	1.03	[0.01, 0.04]	<0.001
Percent female	−0.04	0.96	[−0.06, −0.03]	<0.001
Percent non-English-speaking households	0.03	1.03	[0.01, 0.04]	0.002
Percent has a computer and broadband connection	0	1	[−0.01, 0.01]	0.48
